# Supplementary material for: Acoustically manipulating internal structure of disk-in-sphere endoskeletal droplets
Source: Nat Commun. 2022 Feb 21;13:987. doi: 10.1038/s41467-022-28574-4 (PMC8861019; doi:10.1038/s41467-022-28574-4)
Supplement: Supplementary file 1 — Supplementary Information [file 41467_2022_28574_MOESM1_ESM.pdf]

Supplementary Information

Acoustically Manipulating Internal Structure of Disk-in-Sphere Endoskeletal Droplets

Gazendra Shakya†, Tao Yang†, Yu Gao, Apresio K. Fajrial, Baowen Li, Massimo Ruzzene, Mark A. Borden, and Xiaoyun Ding*

*Corresponding author. Email: xiaoyun.ding@colorado.edu
† These authors contributed equally to this work.

This PDF file includes:

Supplementary Section 1 to 5
Supplementary Figures 1 to 12
Supplementary Table 1
References

Other Supplementary Materials for this manuscript include the following:

Supplementary Movies 1 to 11

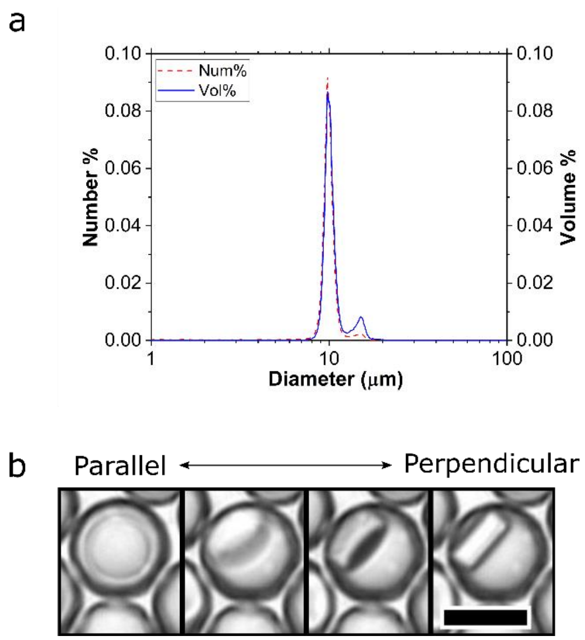

Supplementary Figure 1 : Endoskeletal Droplets. a. Size distribution of the endoskeletal droplets generated using microfluidics (shown in Fig. 1) showing both the number percent (red dashed line) and volume percent (blue solid line). Source data are provided as source data file. **b.** Series of images showing the same endoskeletal droplet with rotating and translating disk from parallel orientation to perpendicular orientation. Scale bar: 10 μm.

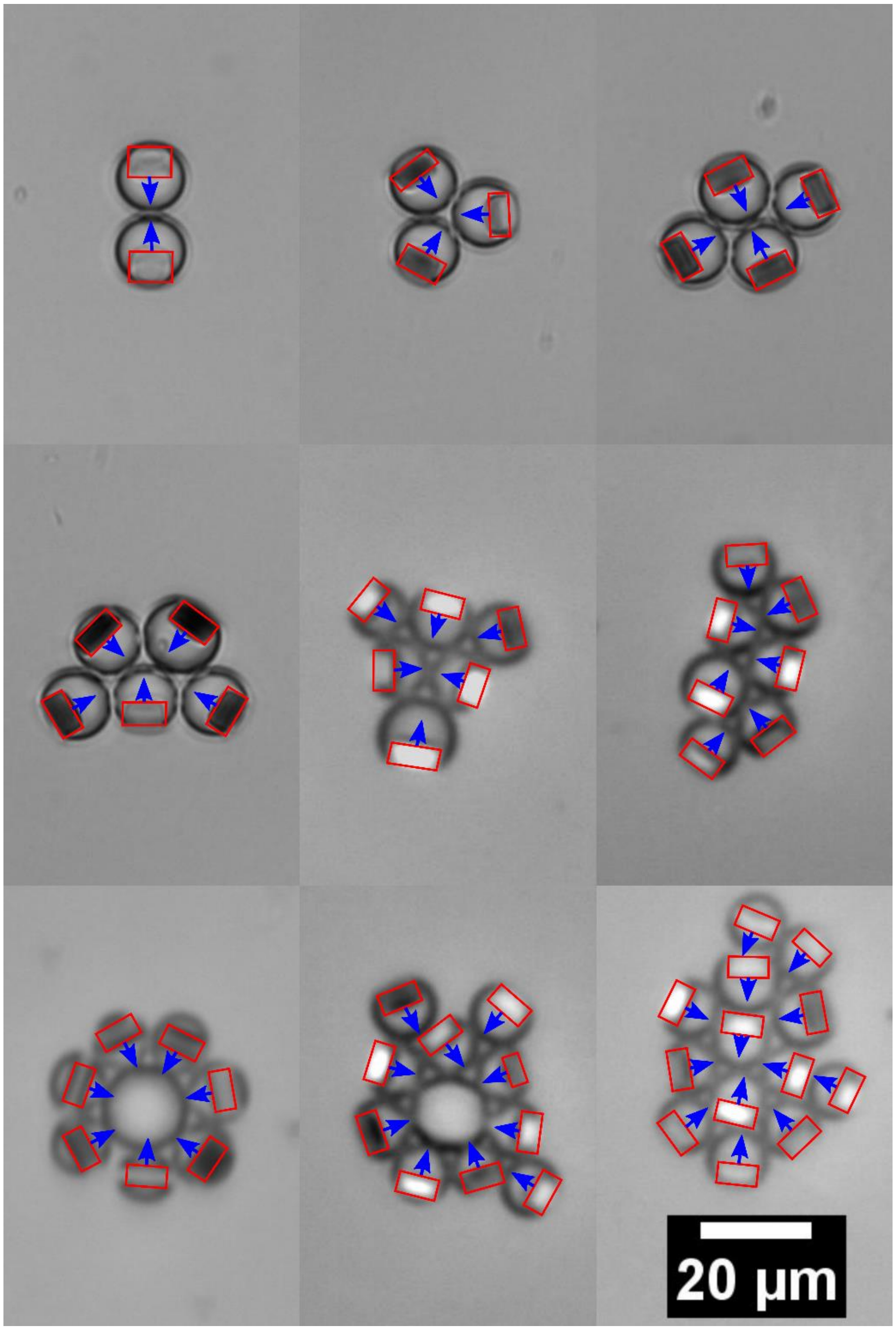

Supplementary Figure 2: Clusters containing different numbers of endoskeletal droplets under SSAW. The disks are oriented in such a way that the normal (blue arrow) drawn from the basal plane of the disk (red rectangle) always points to the centroid of the cluster. This behavior is consistent irrespective of the number of droplets in the cluster.

Supplementary Section 1: Comparison of secondary radiation interactions between two polystyrene particles and PFH droplets

The secondary radiation interaction energy between two PFH droplets on the plane parallel to the wave propagation direction close to anti-nodal line (i.e. Eqn. 4 in the main text) can be obtained when $kh = \pi/2$ is substituted into Eqn. 19 in Silva and Bruus [1]:

$$U_{sec} = \pi E_0 k^3 a^6 \left[\frac{2}{3} f_{1,l/w} f_{0,l/w} \sin(krcos\delta) \, cos\delta \left(\frac{coskr}{(kr)^2} + \frac{sinkr}{kr}\right) - \frac{4}{9} \frac{f_{0,l/w}{}^2 \cos(krcos\delta) \, coskr/kr}{kr}\right] \quad \text{(S1)}$$

For $r \sim a$, i.e. in short range, $kr << 1$, the second term in Eqn. S1 can be neglected and Eqn. S1 can thus be further simplified to Eqn. 4 after small angle approximations and trigonometric function simplifications where:

$$\begin{aligned} \sin(krcos\delta) \, coskr &= \tfrac{1}{2}[\sin(krcos\delta + kr) + \sin(krcos\delta - kr)] \\ &\cong \tfrac{1}{2}[krcos\delta + kr + krcos\delta - kr] = krcos\delta \end{aligned} \quad \text{(S2i)}$$

$$\begin{aligned} \cos(krcos\delta) \, coskr &= \tfrac{1}{2}[\cos(krcos\delta + kr) + \cos(krcos\delta - kr)] \\ &\cong \tfrac{1}{2}\left[1 - \tfrac{1}{2}(krcos\delta + kr)^2 + 1 - \tfrac{1}{2}(krcos\delta - kr)^2\right] \sim 1 \end{aligned} \quad \text{(S2ii)}$$

Similarly, when $kh = 0$ is substituted into Eqn. 19 in Silva and Bruus [1], the secondary interaction between two PS particles can be obtained as:

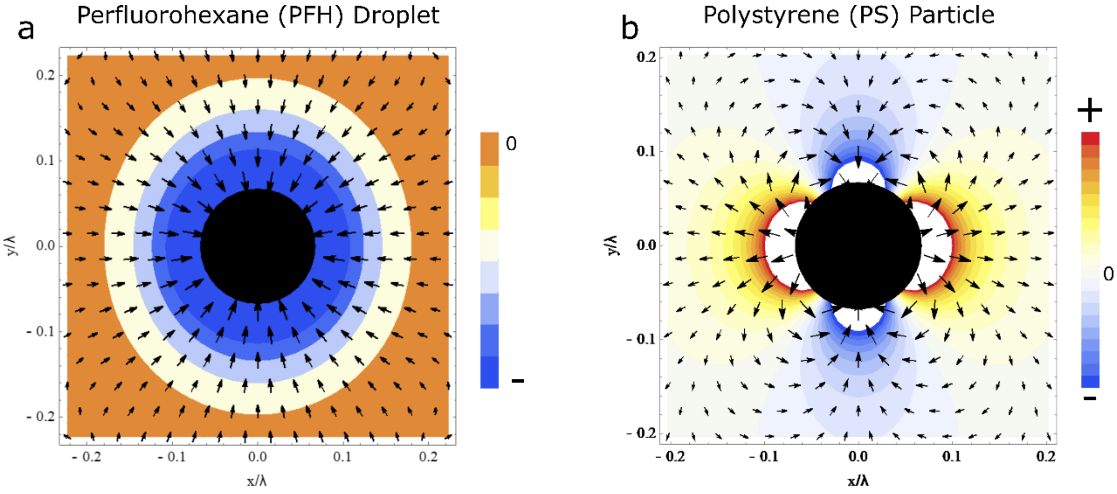

Supplementary Figure 3: PFH vs PS patterning. The acoustic interaction pair potential U_{sec} (Eqn. 4) and $U_{sec}{}'$ (Eqn. S3) (contours) and force $F_{sec} = -\nabla U_{sec}$ (or $F_{sec}{}' = -\nabla U_{sec}{}'$) (arrows) between two PFH droplets (**a**) and PS particles (**b**). Attraction in PS particles is anisotropic and angle dependent (attraction close to 90°, repulsion ~ 0°) whereas attraction in PFH particles is isotropic (attracted from all angles). This results in the formation of clusters for PFH (as seen in Fig. 2h-j, 3c) whereas chains for PS (as seen in Fig. 3d).

$$U_{sec}' = \pi E_0 a^6 f_{1,p/w}{}^2 \frac{1+3\cos 2\delta}{2r^3}, \quad \text{(S3)}$$

which is consistent with Eqn. 22a in Silva and Bruus [1] if $\delta = \pi/2$.

The acoustic interaction pair potential U_{sec} (Eqn. 4) and U_{sec}' (Eqn. S3) (contours in Supplementary Fig. 3) and force $F_{sec} = -\nabla U_{sec}$ (or $F_{sec}' = -\nabla U_{sec}'$) (arrows in Supplementary Fig. 3) between two PFH droplets and two polystyrene particles is thus compared in Supplementary Fig. 3. It is clearly seen for droplets with a negative acoustic contrast factor and a dominant monopole scattering factor, like PFH droplets, the interactions are almost isotropic and thus drive compact cluster formation (Fig. 2h-j, 3c). Conversely, for microparticles with a positive acoustic contrast factor (do not need a dominant dipole scattering factor) like polystyrene particles as the secondary interaction energy is dipolar and highly orientation dependent. The interaction from repulsive to attractive happens at so-called magic angle ~ 54.7° and the lowest energy occurs at $\delta = \pi/2$, resulting in chains as the most stable configurations under 1D standing wave (Fig. 3d). Shorter range and much weaker interactions can also be expected for two polystyrene particles compared to two PFH droplets.

Supplementary Section 2: Numerical simulation on skeletal disk orientations

Finite element simulations were performed using COMSOL (ver 5.0) to find the equilibrium disk orientations for endoskeletal droplet clusters. Here, the Helmholtz equations with endoskeletal droplet cluster configurations were solved numerically. Pressure domain acoustic *acpr* module in COMSOL was used to numerically solve the Helmholtz equation,

$$\nabla^2\phi + k^2\phi = 0 \quad \text{(S4)}$$

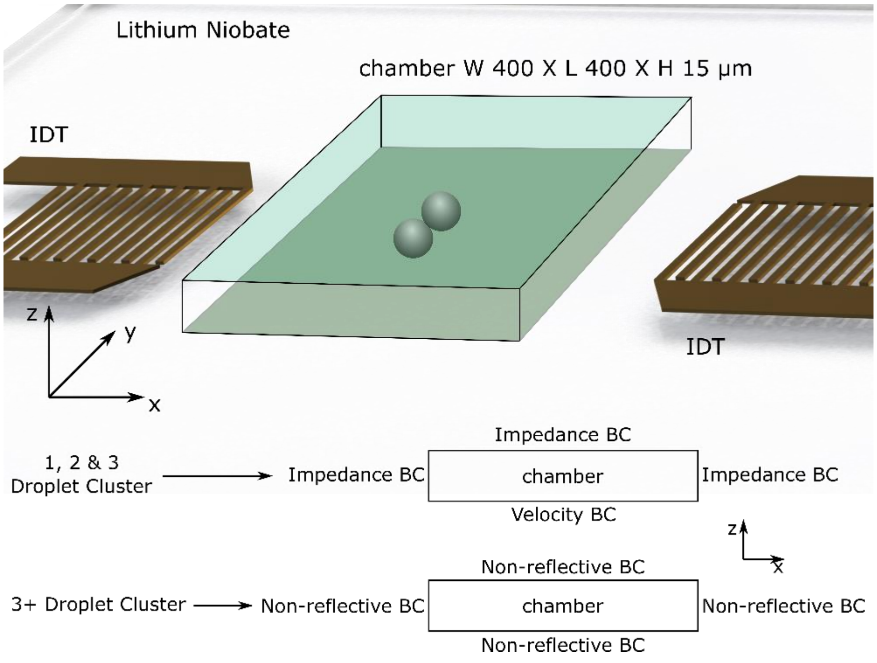

Supplementary Figure 4: COMSOL Schematic. Schematic of the finite element simulation performed in COMSOL showing the boundary conditions used for the simulations.

where ϕ is the particle velocity potential, i.e. $v_1 = \nabla\phi$. The whole simulation domain is composed of W 400 µm (~2λ , 20 MHz)× L 400 µm × H 18 µm (~ 3.6 a) chamber (Supplementary Fig. 4) filled with water and PFH droplets and PFDD disks as separate *pressure acoustic (acpr)* domains. A 1D standing wave was implemented as the background field. Non-reflective boundaries were set for all the chamber surfaces following others [2–4].

For small clusters like monomers and dimers, a velocity boundary condition following Devendra et, al. [5] was applied to the bottom $LiNbO_3$ surface as the leaky wave was taken into consideration. All other surfaces bound by PDMS were taken as impedance boundaries implemented with PDMS acoustic properties due to computer memory constraints. Since the effect of the leaky wave is much less dramatic for clusters that are larger than trimers, only a background 1D standing wave was implemented along with perfectly matched layers for all the boundaries (Supplementary Fig. 4).
The computation mesh is composed of a Free Tetrahedral meshing composing liquid droplets plus inner disks and Boundary layers mesh with 5 boundary layers of a total thickness 0.5 mm composing rectangular channel surfaces. The rest of meshing is confined by a maximum element size of $\lambda_w/5$ as recommended by the COMSOL manual. The maximum element size of Free tetrahedral was chosen as a/6 as justified by the mesh refinement test in Supplementary Fig. 11.

After solving the wave equations along with boundary conditions, the force and torque on an inner disk can be calculated via the integral of surface tensors along a surface enclosing the disk following Bruus [6] as:

$$\boldsymbol{F} = \iint \left(\left(\frac{\langle {p_1}^2 \rangle}{2\rho_l {c_l}^2} - \frac{\rho_l \langle \boldsymbol{v_1} \cdot \boldsymbol{v_1} \rangle}{2} \right) \widehat{\boldsymbol{n}} + \rho_l \langle (\widehat{\boldsymbol{n}} \cdot \boldsymbol{v_1}) \boldsymbol{v_1} \rangle \right) ds \qquad \text{(S5)}$$

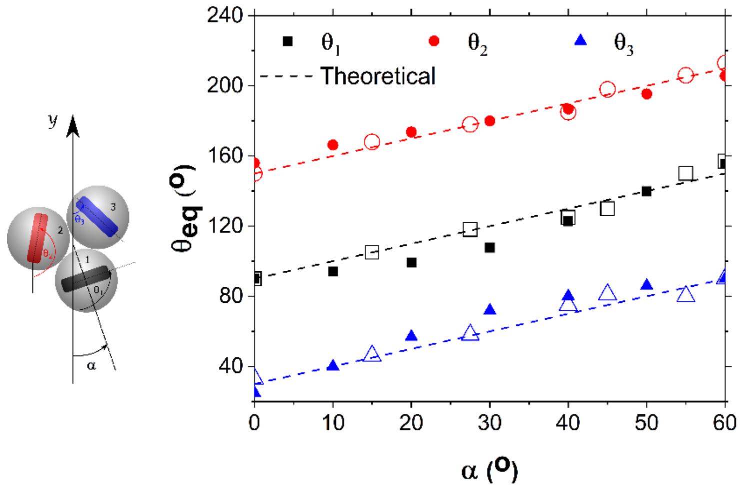

Supplementary Figure 5: Disk angle vs cluster angle. Equilibrium angles (θ) for the ith disk shown for various values for cluster orientation (α). The solid symbols show the calculated angles from the simulations (disk 1 - black square, disk 2 - red circle and disk 3 - blue triangle). The empty symbols show the experimental angles for the respective disks. The dotted line represents predictions based on cluster symmetry, i.e. $\boldsymbol{\theta_{i,eq}} = \frac{5}{6}\boldsymbol{\pi} - \frac{\pi}{3}\boldsymbol{i} + \boldsymbol{\alpha}$

$$\boldsymbol{\tau} = \iint \boldsymbol{r_{so}} \times \left(\left(\frac{\langle p_1{}^2 \rangle}{2\rho_l c_l{}^2} - \frac{\rho_l \langle \boldsymbol{v_1} \cdot \boldsymbol{v_1} \rangle}{2} \right) \hat{\boldsymbol{n}} + \rho_l \langle (\hat{\boldsymbol{n}} \cdot \boldsymbol{v_1}) \boldsymbol{v_1} \rangle \right) ds$$

where $\hat{\boldsymbol{n}}$ is a unit normal vector pointing outwards the surface s, $\boldsymbol{r_{so}}$ the position vector connecting the center of mass of disk/droplet and the point on the surface, p_1 and $\boldsymbol{v_1}$ is obtained from the solved wave equations.

As for the solid disk in a standing acoustic wave, the skeletal disk orientations are also determined by equilibrium zero torque configurations. The calculated torque from Eqn. S5 on i^{th} disk, $\tau_{z,i}$, inside a trimer droplet cluster was found $\tau_{z,i} \propto \sin\left(2\left(\theta_i - \alpha + \frac{\pi}{3}(i-1)\right)\right)$ (Fig. 4a, 4b), where θ_i is the orientation angle of the disk in the ith droplet and antinodal line (positive y axis) whereas α is defined as the angle between the antinodal line and the line joining the centroid and the center of the 1st droplet (which gives us the orientation of the whole cluster). Geometrically, the ith disk equilibrium orientation should thus be $\theta_{i,eq} = \frac{5}{6}\pi - \frac{\pi}{3} i + \alpha$ (confirmed with changing α, Supplementary Fig. 5), i.e. the normal to each disk's basal plane points to the trimer centroid, which is consistent with experimental results (Supplementary Fig. 2, equilibrium θ values for different α shown in Supplementary Fig. 5).

Supplementary Table 1: Equilibrium angles (θ) of the disks in different cluster sizes calculated at α = 0

# of Droplets	θ_1	θ_2	θ_3	θ_4	θ_5	θ_6	θ_7
3	90	155	24	-	-	-	-
4	65	167	65	167	-	-	-
5	51	162	90	130	18	-	-
6	57	160	90	20	123	90	-
7	0	58	122	0	58	122	NA

The same consistent simulation results were also confirmed for all other larger clusters. The equilibrium angles (calculated at α = 0 from zero torque configurations) for cluster with 3 to 7

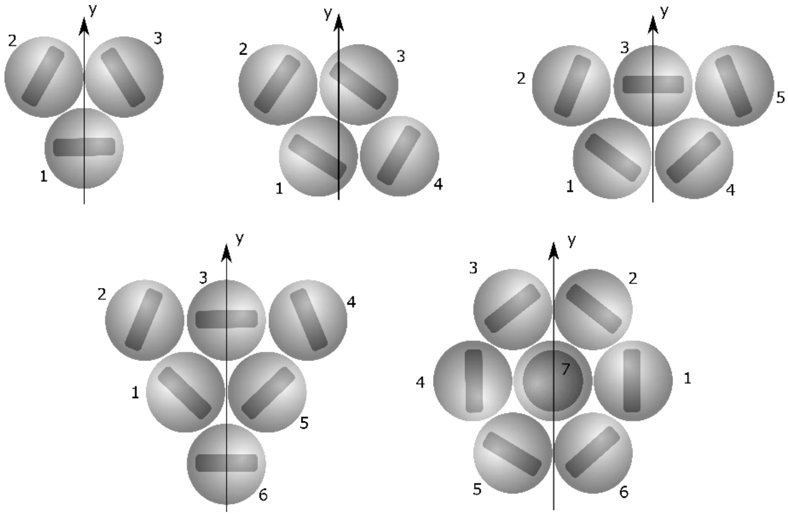

Supplementary Figure 6: Droplet cluster schematic. Schematic of 3 to 7 droplet clusters showing ith droplet. Disk orientations based on numerical simulation and the ith disk angles are tabulated in Table S1. Compare with experimental disk orientations in Fig. 2e-g and Supplementary Fig. 4.

droplets are listed in the Supplementary Table 1 and their schematic is shown in Supplementary Fig. 6. These disk angles are consistent with the orientation of the disks seen in experiments (Fig. 2e-g, Supplementary Fig. 2) for clusters with same number of droplets.

Supplementary Section 3: Dynamic Simulations

Note that torque calculations in Fig. 4a and Fig. 4b (also Supplementary Table 1, Supplementary Fig. 5 and Supplementary Fig. 6) assume that disks are at the droplet centers and non-moving. In experiments, because of its positive contrast factor with respect to the surrounding PFH environment, disks are pushed to the edges of the droplets (as seen in experimental results Fig. 2d-g, Supplementary Movie 1). Thus, disk dynamics simulations were also carried out. Each disk was represented by two-layer hexapolarly aligned ($N = 2 \times 19 = 38$) smaller spheres radius (a_p = 1.34 μm with same disk density) with a rigid Hooken spring connected between two neighboring spheres (Supplementary Fig. 7).

The external forces on each small sphere include pointwise radiation force $\boldsymbol{F_{pri}} = -\nabla \mathrm{U_{rad}}$, which can be derived from Eqn.1 once the wave equation with fixed droplet position is solved, and the drag force $\boldsymbol{F_{dp}} = -6\pi\eta a_p \boldsymbol{v_p}$, where η is PFH liquid viscosity and $\boldsymbol{v_p}$ is the small sphere instantaneous velocity. The inter-sphere Hooken spring force is given as: $\boldsymbol{F_s} = -k_s \sum_{i=1}^{N} (|\boldsymbol{r} - \boldsymbol{r}_i| - r_0) \frac{\boldsymbol{r}-\boldsymbol{r_i}}{|\boldsymbol{r}-\boldsymbol{r_i}|}$) and in order to confine spring forces only between neighboring spheres, a cutoff length r_c = 2.8 a_0 was applied. The instantaneous velocity on the ith small sphere $\boldsymbol{v_{p,i}}$ can be solved via a set of ODE equations:

$$m_p \frac{d\boldsymbol{v_{p,i}}}{dt} = \boldsymbol{F_{pri}} + \boldsymbol{F_{dp,i}} + \boldsymbol{F_{s,i}} \ (r_{ij} < r_c) \quad \text{(S6)}$$

where $m_p = \rho_s \frac{4}{3}\pi {a_0}^3$ is the mass of each small sphere. Thus, the whole disk trajectory and orientation at any moment can be obtained afterwards. As seen from the simulated disk dynamics inside a trimer cluster (Supplementary Movie 4), disks standing perpendicular to the substrate were pushed to the edge of the enclosing droplet with long axis parallel to the contact line, consistent

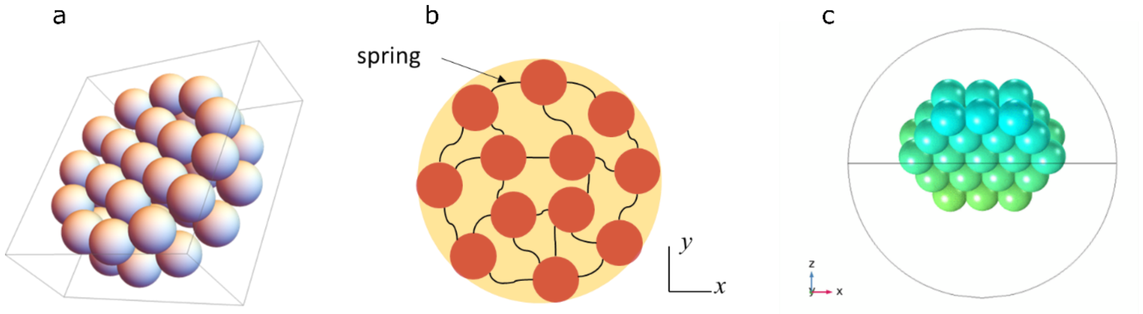

Supplementary Figure 7: Schematic of the disk for dynamic simulations. a. Each disk is represented by two layered hexapolarly aligned smaller spheres. **b.** Each small sphere is connected with a rigid Hooken spring. **c.** Schematic of the constructed disk inside the droplet.

with experimental results. The consistent orientations with equilibrium torque calculations also indicate symmetric field distributions along axes of symmetry of the cluster.

Supplementary Section 4: Contribution of Leaky Wave

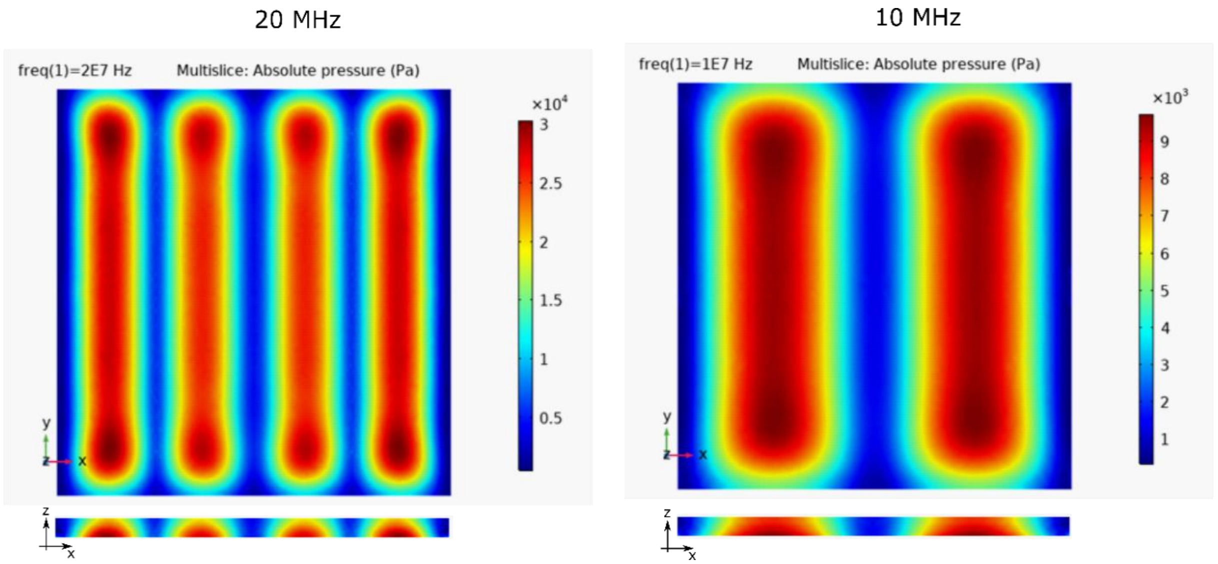

Supplementary Figure 8: Leaky wave pressure distributions. Pressure amplitude distributions on a chamber (400 X 400 X 15 μm) formed by 1D standing waves (acoustic waves moving in positive and negative x-axis). Fully formed standing waves are generated in the x-axis. Since the channel height is much smaller than the wavelength, quasi-standing waves are formed in the z axis. This is seen for both 20 MHz and 10 MHz wave.

For the real case, surface acoustic wave is more complicated when the leaky wave from lithium niobite substrate moving into fluid medium is taken into consideration. Following the numerical simulation process developed by Nama, et al.[7], Devendra et al.[5], and Barnkob, et al.[8], a velocity boundary condition (Eqn. 12 in Ref.[2]) was set on the bottom PDMS surface, while acoustic impedance boundaries on all other surfaces (Supplementary Fig. 4). The calculated pressure distribution without droplets is shown in Supplementary Fig. 9. It's clearly seen that in addition to a standing wave in x axis, a sophisticated pressure distribution along z axis is observed. As in our case the channel height is confined at a height of ~3a, a quasi-standing wave without a fully developed period is observed as in Supplementary Fig. 8. Since particle velocity $\boldsymbol{v_1}$ inside droplets is the sum of primary or incident particle velocity $\boldsymbol{v_{pri}}$ and particle velocity from scattering wave $\boldsymbol{v_{sec}}$ and related with acoustic pressure as:

$$\boldsymbol{v_1} = \boldsymbol{v_{pri}} + \boldsymbol{v_{sec}} = \frac{\nabla p_1}{i\rho_l\, c_l} = \frac{1}{i\rho_l\, c_l}(\nabla p_{pri} + \nabla p_{sec}) \qquad \text{(S7)}$$

where p_{pri}/p_{sec} is the incoming/scattered acoustic pressure, i is the imginary unit. As $\nabla p_{pri} = \nabla p_{in} = -p_0(\sin(k_{LN}x)\cos(k_w \sin\ \theta_R\ z\ )\hat{\mathbf{x}} + \cos(k_{LN}x)\sin(k_w \sin\ \theta_R\ z\ )\hat{\mathbf{z}})$ and incident particle velocity at droplet center can be approximated as $\boldsymbol{v_{pri}} = \frac{1}{i\ \rho_l\ c_l}\nabla p_{pri} =$

$\frac{p_0}{i\,\rho_l\,c_l}\sin(k_w \sin\ \theta_R\, a\,)\,\hat{\mathbf{z}} \sim \frac{p_0}{i\,\rho_l\,c_l} k_w \sin\ \theta_R\, a\,\hat{\mathbf{z}}$, where $x = 0$ (antinodal line), $z = a$ and $k_w \sin\ \theta_R\, a \ll 1$.

As the monopole term is dominant over dipole term in our system, secondary scattering velocity potential $\phi_{sec} = \frac{p_{sec}}{i\rho_l\,c_l}$ can be simplified from Eqn. S6 in [4] as:

$$\phi_{sec} \sim \phi_{sec,0} = i\omega f_{0,l/w} \frac{a^3 p_{in}(\boldsymbol{r}_s)}{3\rho_l {c_l}^2} \frac{e^{ik_w r}}{r} = i f_{0,l/w} \frac{a^3 p_0 k_l}{3\rho_l c_l} \frac{e^{ik_w r}}{r} \tag{S8}$$

where $\boldsymbol{r_s} = (0,0,a)$ is the postion vector of the scatterer and $p_{in}(\boldsymbol{r}_s) = p_0 \cos(k_w \sin\ \theta_R\, a) \sim p_0$. Thus secondary particle velocity $\boldsymbol{v_{sec}}$ can be simplfied as:

$$\boldsymbol{v_{sec}} = \nabla\phi_{sec} = -\, i f_{0,l/w} \frac{a^3 p_0 k_l}{3\rho_l c_l} \frac{1}{r^2} \hat{\mathbf{r}} \tag{S9}$$

The secondary particle velocity therefore is purely radial and is along the y-axis in the case of dimer droplets.

For larger clusters like a trimer, if assuming the multibody scattering as the sum of binary scattering from neighboring droplets,

$$\boldsymbol{v_1} = \boldsymbol{v_{pri}} + \sum \boldsymbol{v_{sec,ij}} \tag{S10}$$

Since from Eqn. S9, the seconary scattering velcotiy $\boldsymbol{v_{sec}}$ is along the radial direction, the sum of all secondary scattering from neighboring droplets on any droplet thus is along the line connecting droplet centroid to the whole cluster centroid (see Supplementary Fig. 9 for scehmatics for a trimer cluster). More importantly, the amplitude of summed secondary scattering velocity becomes larger as: $|\boldsymbol{v}'_{\boldsymbol{sec}}| = \sqrt{3}|\boldsymbol{v_{sec}}|$. So total particle velocity aligns with x axis with an angle atan(1/3)~18.4 ° and disk will align more steeper with *xy* plane compared with a dimer cluster.

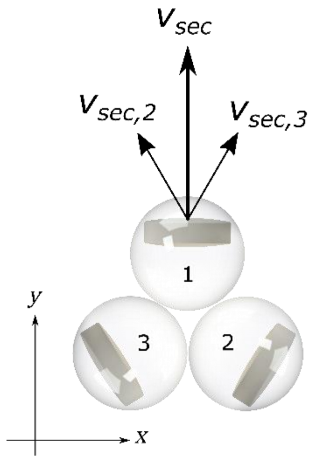

Supplementary Figure 9: Sum of secondary scattering velocity. Schematic of a cluster with three droplets showing the effects of secondary radiation force on disk 1 from droplet 2 and 3.

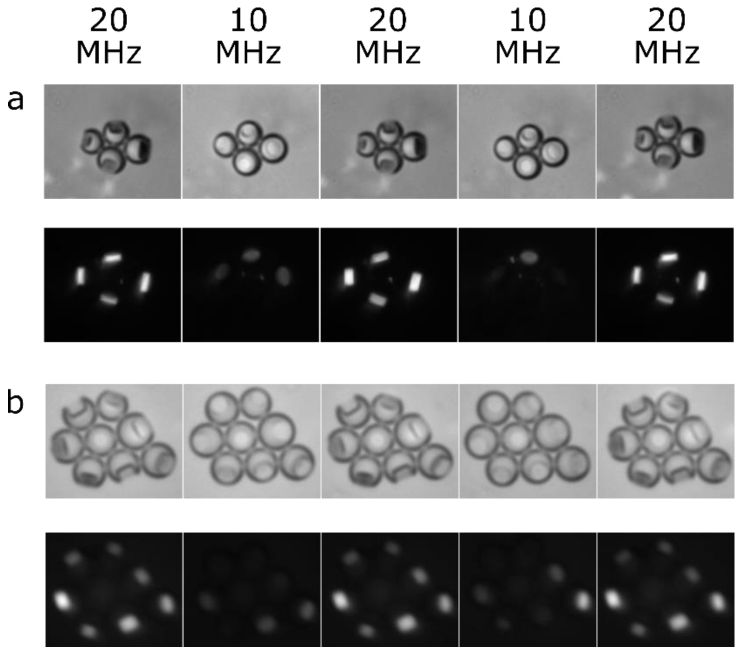

Supplementary Figure 10: External control of disk orientation. Series of images showing the corresponding switching between a perpendicular to a parallel orientation of the disks along with changing frequencies from 20 to 10 MHz and vice versa for a 4 droplet cluster (**a**) and a 8 droplet cluster (**b**) (Movie S11 for corresponding video). Top figures show brightfield images whereas bottom figures show CPM images.

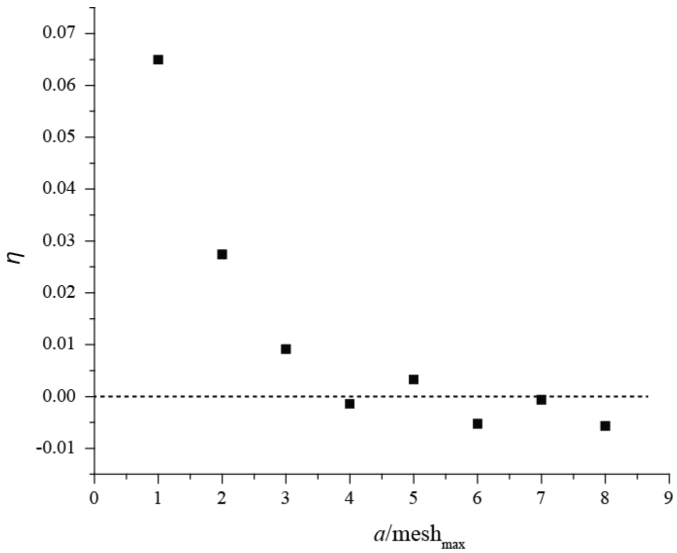

Supplementary Figure 11: Mesh refinement test, $\eta = (p_{1,max} - p_{1,max,ref})/p_{1,max,ref}$ vs. a/mesh$_{max}$. For simplicity we compared the maximum pressure amplitude inside the droplet of a solution at varying maximum mesh element size $\mathrm{mesh}_{\mathrm{max}}$ compared with that at finest mesh element ($a/\mathrm{mesh}_{\mathrm{max}} = 9$).

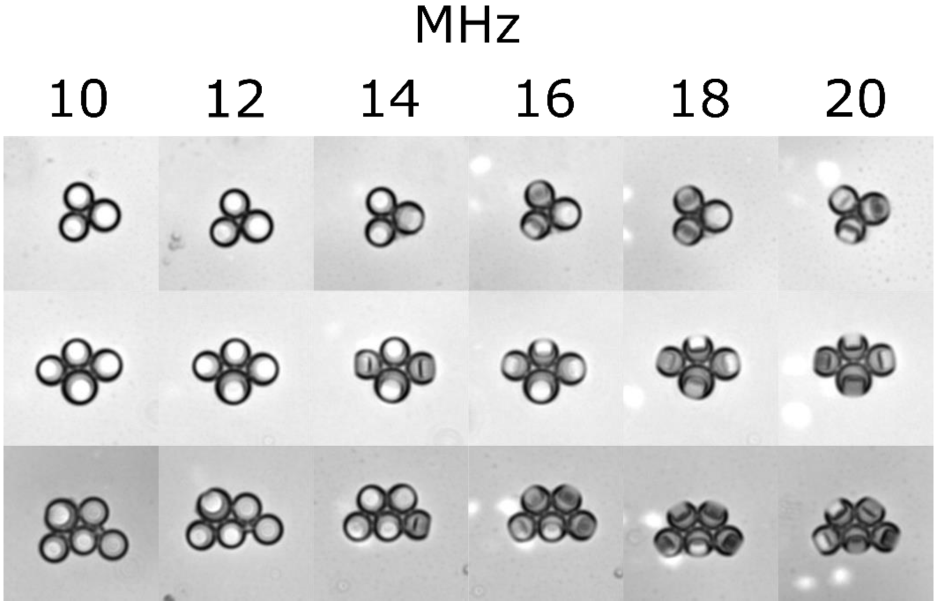

Supplementary Figure 12: Intermediary frequencies. Series of images with 3 droplets cluster at top, 4 droplet cluster in second row and 5 droplet cluster at the bottom shown at different intermediary frequencies. Note that larger droplet clusters require smaller frequencies for the disks to be perpendicular.

Supplementary Section 5: Intermediary Frequencies

Note that the current work focuses on the droplet aggregation and disk orientation at a frequency of 10 and 20 MHz as these two frequencies show us the two extremes of the disk orientation

behavior. At 10 MHz, even very large clusters show parallel orientation (Fig 5a) whereas in 20 MHz, 2 droplets clusters show the threshold transition between the parallel and perpendicular orientation of the disks (Fig 2d). This perpendicular orientation is brought about by the effect of the secondary radiation force. The effect of the secondary radiation force can be increased in two ways. First by increasing the frequency (equation 4), and second by increasing the number of droplets in a cluster (equation S10). Hence, smaller cluster of droplets would require higher frequencies for the disks to be perpendicular whereas disks in larger cluster of droplets would require lower frequencies for the disks to be perpendicular. To look into the effects of the number of droplets we performed similar experiments at various intermediary frequencies (12, 14, 16 and 18 MHz) using a chirped IDT device. The results are shown in Supplementary Figure 12 where we observed that for a 3-droplet cluster, most disks are perpendicular at 18-20 MHz, for a 4-droplet cluster, most disks are perpendicular at 16-18 MHz and finally for a 5-droplet cluster, most disks are perpendicular at 14-16 MHz.

Since our goal with this paper was to show on-demand flipping of the disks from parallel to perpendicular, 10 and 20 MHz works well as it shows the two extremes of the disk arrangement behaviors for all the droplet clusters. Moreover, it is obvious, based on the investigation of the principles behind such phenomenon in this work, that disk orientation transition occurs between 10-20 MHz, as shown in the additional experimental observation in Supplementary Figure 12.

References

1. Silva, G. T. & Bruus, H. Acoustic interaction forces between small particles in an ideal fluid. *Physical Review E* **90**, (2014).
2. Glynne-Jones, P., Mishra, P. P., Boltryk, R. J. & Hill, M. Efficient finite element modeling of radiation forces on elastic particles of arbitrary size and geometry. *The Journal of the Acoustical Society of America* **133**, 1885–1893 (2013).
3. Garbin, A. *et al.* Acoustophoresis of disk-shaped microparticles: A numerical and experimental study of acoustic radiation forces and torques. *The Journal of the Acoustical Society of America* **138**, 2759–2769 (2015).
4. Simon, Andrade, Desmulliez, Riehle, & Bernassau. Numerical Determination of the Secondary Acoustic Radiation Force on a Small Sphere in a Plane Standing Wave Field. *Micromachines* **10**, 431 (2019).
5. Devendran, C., Albrecht, T., Brenker, J., Alan, T. & Neild, A. The importance of travelling wave components in standing surface acoustic wave (SSAW) systems. *Lab on a Chip* **16**, 3756–3766 (2016).
6. Bruus, H. Acoustofluidics 7: The acoustic radiation force on small particles. *Lab on a Chip* **12**, 1014 (2012).
7. Nama, N. *et al.* Numerical study of acoustophoretic motion of particles in a PDMS microchannel driven by surface acoustic waves. *Lab on a Chip* **15**, 2700–2709 (2015).
8. Barnkob, R. *et al.* Acoustically Driven Fluid and Particle Motion in Confined and Leaky Systems. *Physical Review Applied* **9**, (2018).
