# Supplementary material for: Acoustically manipulating internal structure of disk-in-sphere endoskeletal droplets
Source: Nat Commun. 2022 Feb 21;13:987. doi: 10.1038/s41467-022-28574-4 (PMC8861019; doi:10.1038/s41467-022-28574-4)
Supplement: Supplementary file 3 — Description of Additional Supplementary Files [file 41467_2022_28574_MOESM3_ESM.pdf]

Description of Additional Supplementary Files

File Name: Supplementary Movie 1
Description: Video showing disk interactions when standing SAW is turned on. Note that when standing SAW is turned on, the solid disks start orienting when it comes close to other droplets. Also note the change in orientation of the disks as the size of the droplet cluster changes

File Name: Supplementary Movie 2
Description: For a single droplet, when the SAW is turned on, the solid disk flips up to the top and orients parallel to the substrate (parallel to xy plane).

File Name: Supplementary Movie 3
Description: For a cluster with two droplets, the droplets first form the cluster when the SAW is turned on and then the disks orient such that they are they are midway between the parallel and perpendicular orientations (~ 45o from the xy plane).

File Name: Supplementary Movie 4
Description: Dynamic simulations of cluster containing 3 droplets at 20 MHz shows the disks being pushed to the edges of the cluster as seen in experiments (Fig. 2e). Left shows the xy view and right shows the xz view

File Name: Supplementary Movie 5
Description: Dynamic simulation of a single droplet. Because of the primary radiation force (and the absence of secondary radiation force from neighboring droplets) from the traveling wave in z axis, the disks float up to the top and are parallel to the surface (xy plane). Left shows the xy view and right shows the xz view.

File Name: Supplementary Movie 6
Description: Dynamic simulation of a 2-droplet cluster at 10 MHz (top) and 20 MHz (bottom). Here, left column is the xy view and the right column is the yz view. Since the secondary radiation force is more sensitive to frequency, decreasing frequency would decrease the effect of the secondary radiation force. Hence, the disks are more parallel to the surface for 10 MHz than for 20 MHz.

File Name: Supplementary Movie 7
Description: Video showing disk orientation when 10 MHz standing SAW is turned on. Note that the disks orient parallel to the surface (as opposed to perpendicular for 20 MHz in Supplementary Movie 1).

File Name: Supplementary Movie 8
Description: Dynamic Simulations of a 3-droplet cluster showing the disk orientations at 10 MHz (top) and 20 MHz (bottom). The left column is the xy view and the right column is the xz view.

File Name: Supplementary Movie 9
Description: The orientation of the disk can be tuned by changing the frequencies. The video shows a 3-droplet cluster where the frequency of the acoustic waves is switched back and forth between 10 and 20 MHz.

File Name: Supplementary Movie 10
Description: The orientation of the disks in a 3-droplet cluster can be tuned by changing the frequencies back and forth between 10 and 20 MHz. Under CPM, disks in perpendicular orientation (@20 MHz) are clearly seen whereas disks in parallel orientation (@10 MHz) are not seen.

File Name: Supplementary Movie 11
Description: The orientation of the disks can be tuned by changing the frequencies back and forth between 10 and 20 MHz in 4-droplet cluster and 8-droplet cluster.
